# 5G Electromagnetic Radiation Attenuates Skin Melanogenesis In Vitro by Suppressing ROS Generation

**DOI:** 10.3390/antiox11081449

**Published:** 2022-07-26

**Authors:** Kyuri Kim, Young Seung Lee, Nam Kim, Hyung-Do Choi, Kyung-Min Lim

**Affiliations:** 1 College of Pharmacy, Ewha Womans University, Seoul 03760, Korea; kyuri@ewhain.net; 2 Radio & Satellite Research Division, Electronics and Telecommunications Research Institute, Daejeon 34129, Korea; lys009@etri.re.kr (Y.S.L.); choihd@etri.re.kr (H.-D.C.); 3 Department of Computer and Communication Engineering, Chungbuk National University, Cheongju 28644, Korea; namkim@chungbuk.ac.kr

**Keywords:** non-ionizing radiation, electromagnetic waves, 5G, skin pigmentation, melanogenesis

## Abstract

Recently, the impacts of 5G electromagnetic radiation (EMR) with 28 GHz on human health have been attracting public attention with the advent of 5G wireless communication. Here, we report that 5G (28 GHz) EMR can attenuate the skin pigmentation in murine melanoma cells (B16F10) and a 3D pigmented human epidermis model (Melanoderm™). B16 cells were exposed to 5G (28 GHz) with or without α-MSH for 4 h per day. Interestingly, 5G attenuated α-MSH-induced melanin synthesis. Fontana–Masson staining confirmed that the dendritic formation of α-MSH stimulated B16 cells was diminished by 5G exposure. To confirm the anti-melanogenic effect of 5G EMR, MelanoDerm™ was irradiated with 5G at a power intensity of 10 W/m^2^ for 4 h a day for 16 days and melanin distribution was detected with Fontana–Masson staining, which supported the anti-melanogenic effect of 5G EMR. Consistently, 5G EMR suppressed α-MSH induced upregulation of melanogenic enzymes; tyrosinase, TRP-1, and TRP-2. Of note, 5G EMR attenuated ROS production stimulated by α-MSH and H_2_O_2_, suggesting that 5G EMR may dissipate ROS generation, which is pivotal for the melanin synthesis. Collectively, we demonstrated that 5G EMR can attenuate skin pigmentation by attenuating ROS generation.

## 1. Introduction

With the advent of the fifth generation (5G) network era, 5G communication devices with 28 GHz frequency bandwidth are being widely used in our everyday lives. Also, the application of 5G has been extended to various kinds of electronic devices that integrate 4G, Wi-Fi, and millimeter waves (mmW), as well as to other wireless appliances [1]. However, the widespread use of 5G communication devices has been raising huge public concern due to a serious lack of information on its safety to human health and the environment [2,3]. In particular, the impact of 5G electromagnetic radiation (EMR) on human skin has been drawing attention [4,5,6], since the skin interfaces between the body and the external environment and serves as the primary target tissue for EMR from the environment. Indeed, the EMR such as ultraviolet, visible light like blue light and infrared radiation affect the skin physiology significantly [7,8,9,10], signifying that 5G EMR may also have effects on the skin. Also, it is highly probable that the skin may be the primary target tissue of 5G EMR since 28 GHz EMR from the environment cannot penetrate into our body deeper than 2 mm [11].

Of the most distinguishable changes to the skin after the exposure to UV radiation or blue light is the skin pigmentation, i.e., the accumulation of melanin in the skin epidermis. UV, and blue light irradiation of the skin can induce hyperpigmentation through activating melanogenesis [12,13,14]. Melanin is physiologically synthesized by melanocytes residing in the stratum basale of the skin epidermis. It is transported in the form of melanosomes to keratinocytes differentiating from the basal layer to the upper layers of the skin [15,16]. More than 100 genes participate in the skin pigmentation pathway, including hormones, enzymes, transcription factors, autocrine and paracrine factors, transporters and receptors. In the melanosomes, melanin is synthesized and matured from l-tyrosine by tyrosinase, tyrosinase-related protein 1 (TRP-1), and tyrosinase-related protein 2 (TRP-2). During the synthesis of melanin, reactive oxygen species are generated, which is essential for the progression of melanin synthesis. Rab27a, Mlph, and MyoVa, which form a tripartite complex that is essential for melanosome distribution [17], are also important for skin pigmentation by facilitating the transport of melanosomes into keratinocytes.

We previously studied whether the exposure of either LTE (1.762 GHz) or 5G (28 GHz) EMR could induce skin hyperpigmentation under normal smartphone use conditions [18]. We found that neither LTE nor 5G EMR alone induced melanin synthesis. Interestingly, we discovered that mRNA expression levels of TYR and TRP-1 were slightly affected even though macroscopic skin hyper-pigmentation was not observed, suggesting that 5G EMR may affect the melanogenesis by itself. Here, we examined the effect of 5G (28 GHz) EMR with a daily exposure time of 4 h, which was reported to be the average time spent daily on a smartphone phone use [19] at a power density of 10 W/m^2^. We used a specially designed in vitro 5G exposure system on α-melanocyte stimulating hormone, α-MSH stimulated melanogenesis to get an insight into the impact of 5G EMR on the skin health.

## 2. Materials and Methods

### 2.1. Materials and Reagents

B16F10, a murine melanoma cell line from C57BL/6 mice, was purchased from ATCC (Manassas, VA, USA). B16F10 cells were maintained under standard culture conditions with Dulbecco’s Modified Eagle’s Medium (DMEM) supplemented with antibiotics (100 U/mL of penicillin A and 100 μg/mL of streptomycin) and 10% fetal bovine serum (FBS) at 37 °C in a CO_2_ incubator. At 80% cell confluence, B16 cells were detached with a solution of 0.05% trypsin (Hyclone, South Logan, UT, USA) and seeded for further use.

### 2.2. In Vitro 5G Exposure System

**Scheme 1:**
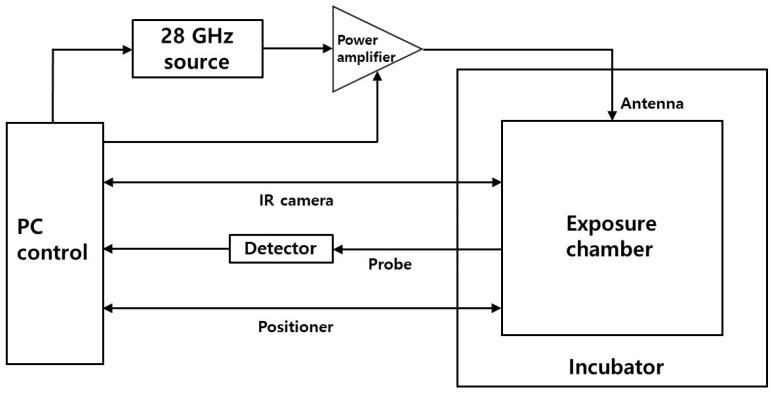
Schematic diagram of 5G exposure system.

An in vitro 5G exposure system for 28 GHz experiments was designed and made for this study (Scheme 1). The “all-in-one” system integrates all system components into a single unit. The exposure chamber was designed according to the previous studies to achieve field uniformity [20,21]. Actual uniformity inside the chamber was checked by planar measurements. Cells exposed were located at a distance of 140 mm from an antenna with an input power of 0.4 W according to the relation between the intensity and the input power inside the chamber [21]. To suppress thermal effects during exposures, especially for high power conditions, temperature was regulated through control of air-flow rates of the incubator according to the cell temperatures measured by the infrared (IR) camera via real-time feedback. All the experimental data were recorded and monitored with a personal computer during exposures. We used a graphical user interface (GUI) of the control software (SW) to easily establish all the operating conditions.

### 2.3. Melanin Content Assay and Cell Viability Assay (MTT)

The B16F10 cells were seeded into a 48-well plate, then exposed to 5G (28 GHz) at a power intensity of 10 W/m^2^ for 4 h per a day either with or without α-MSH treatment. Analysis of melanin content depending on incubation time after EMR exposure was assessed by measuring the cell lysis solution with 1N NaOH at absorbance at 405 nm using an ELISA reader. To identify cytotoxicity 5G exposure on skin pigmentation, B16 melanoma cells were seeded into a 48-well plate and the cells were exposed to 5G (28 GHz) for 4 h per a day for the indicated days. A 3-(4,5-dimethylthiazol-2-yl)-2,5-diphenyltetrazolium bromide (MTT) [22] assay was employed to evaluate the cytotoxicity of 5G exposure on B16F10 cells. B16F10 cells after treatment were incubated with 0.25 mL of 0.5 mg/mL 3-(4,5-dimethylthiazol-2-yl)-2,5-diphenyltetrazolium bromide (MTT) solution in DMEM for 2 h at 37 °C. The resulting blue formazan was dissolved in 0.25 mL of DMSO by rocking for 30 min and 150 μL of supernatant underwent an optical density measurement at 540 nm. All measurements were done in triplicate or more.

### 2.4. Fontana–Masson Staining and Analysis

Fontana–Masson stain kit was purchased from ScyTek Laboratories (Logan, UT, USA). The Fontana–Masson stain was used for histological visualization of melanocyte dendrites. Cells were seeded into an 8-well chamber slide and incubated for 24 h. Then cells were exposed to 5G (28 GHz) at the power intensity of 10 W/m^2^ for 4 h/day either with or without α-MSH treatment. Cell staining was performed according to the indicated incubation time (4 h and 18 h). Cell fixation was proceeded with EtOH (95%) and glacial acetic acid (5%), and then warm ammoniacal silver solution was added to the slide and incubated for 30 min until cell became yellowish/brown in color. Cells were rinsed with distilled water twice, then gold chloride solution (0.2%) was added for 30 s. Another cell wash was performed, and sodium thiosulfate solution (5%) was added for 1 min. Then cells were rinsed for 2 min in running tap water followed by rinsing with distilled water twice. Nuclear fast red solution was added to cells for 5 min, and then cells were rinsed for 2 min in running tap water followed by rinsing with distilled water twice. Cells were dehydrated very quickly in 3 changes of fresh absolute alcohol. The slide was mounted in synthetic resin. The resulting cells were pictured using NIS_BR_Ver 51100 and a Nikon Eclipse Ts2 microscope (X400, eclipse Ts2R, Nikon, Tokyo, Japan). Ratio of activated melanocytes over total cell was analyzed by QuPath 0.3.2 software [23], which is intended to be used for quantitative and bioimage analysis. Total cells and activated melanocytes were counted by a cell counting tool, and the ratio was carried out by activated cells over total cells. Here, activated melanocytes were counted based on the FM staining. Length of activated dendrites were measured by straight lines in Image J. All measurements were performed in triplicate.

### 2.5. Intracellular Reactive Oxygen Species (ROS) Level

ROS generation was measured by 2′,7′-dichlorofluoresceindiacetate (DCF-DA)-enhanced fluorescence assay (Eugene, OR, USA). B16F10 cells were exposed to 5G (28 GHz) at the power intensity of 10 W/m^2^ for 4 h per a day either with or without α-MSH treatment. For a positive control, cells were treated with 1 mM H_2_O_2_ for 1 h and 30 μM ascorbic acid for 4 h before staining. The resulting cells were pictured using NIS_BR_Ver 51100 and a Nikon Eclipse Ts2 microscope (Melville, NY, USA).

### 2.6. RNA Sample Preparation

After treatment, B16F10 cells were washed twice with phosphate-buffered saline and were lysed using Trizol (Invitrogen, Carlsbad, CA, USA). Chloroform was added and samples were centrifuged at 12,000 rpm for 10 min. The resulting aqueous phase was mixed with isopropanol and RNA were collected as pellets after centrifugation at 12,000 rpm, 15 min, 4 °C. RNA pellets were washed with 70% ethanol and dissolved in RNase-free, diethyl pyrocarbonate (DEPC)-treated water (Waltham, MA, USA). The yield of RNA was estimated by measuring the optical density at 260 nm with a NanoDrop 1000 spectrophotometer (NanoDrop technologies, Wilmington, DE, USA).

### 2.7. Real-Time Polymerase Chain Reaction (Real-Time PCR) Assay

Relative mRNA expression level of respective gene was measured by quantitative real-time PCR. cDNA was synthesized from 1250 ng of total RNA with oligo(dT) (Bioelpis, Seoul, Korea). SYBR Green PCR master mix was used in each reaction under a StepOnePlus^TM^ Real-time PCR machine (Applied Biosystems, Warrington, UK). The primer sequences were as follows: forward tyrosinase, 5′-GGGCCC AAA TTG TAC AGA GA-3′; reverse tyrosinase, 5′-ATG GGT GTT GAC CCA TTGTT-3′; forward TRP-1, 5′-GTT CAA TGG CCA GGT CAG CA-3′; reverse TRP-1, 5′-CAGACA AGA AGC AAC CCC GA-3′; forward TRP-2, 5′-TCC AGA AGT TTG ACA GCC C-3′; reverse TRP-2, 5′-GGA AGG AGT GAG CCA AGT TAT G-3′; forward Rab27a 5′-CCA GAG GGC AGT GAA AGA GG-3′, reverse Rab27a 5′-CCG CTT CAT GAT CAG GTC CA-3′; forward Mlph 5′-AGCCCCTCAACAGCAAAAA-3′, reverse Mlph 5′-TTCCTCAAAGTCCACATCTCG-3′; forward MyoVa 5′-GCGCCATCACCCTAAACA-3′, reverse MyoVa 5′-CCAGTTGACTGACATTGTACCTG-3′. Cycling parameters for PCR reaction were 50 ~55 °C for 2 min, 95 °C for 10 min, 40 cycles of 95 °C 15 s, and 50 °C 1 min.

### 2.8. Evaluation of Skin Pigmentation in Melanoderm™, 3D Pigmented Human Epidermal Skin Model

Melanoderm™ was purchased from MatTek Corporation (Ashland, MA, USA). On arrival, the skin model was stabilized in a humidified 37 °C, 5%, CO_2_ incubator overnight (18–24 h) prior to the experiment. The skin tissues were exposed to a 5G signal (28 GHz) for 14 days, while the sham control skin tissues were incubated in a standard incubator. On the last day of the experiment, the tissues were fixed in 10% Formalin and then the histological examination was undertaken. For the histological processing, all tissues were fixed in 4% phosphate-buffered formalin (PFA) for 24 h under gentle shaking. Fixed tissues were paraffin-embedded and cut into 5-mm sections using a microtome (RM2335, Leica, Wetzlar, Germany). Hematoxylin and eosin (H&E) staining was done a day after sectioning. Paraffin sections were deparaffinized and rehydrated in descending ethanol concentrations. Next, sections were stained first with 0.1% Mayer’s hematoxylin for 10 min and then 0.5% eosin in 95% EtOH. After H&E staining, the sections were immediately and sequentially washed as follows: dipping in distilled H_2_O until eosin stops streaking, dipping in 50% EtOH 10 times, dipping in 70% EtOH 10 times, incubating in 95% EtOH for 30 s, and then in 100% EtOH for 1 min. Then, samples on slides were covered with the mounting solution (6769007, Thermo Scientific, Waltham, MA, USA) and examined under a light microscope (BX43, OLYMPUS, Shinjuku, Japan). For Fontana–Masson’s argentaffin staining, paraffin sections were incubated with ammoniacal silver nitrate for 1 h at 60 °C, followed by washing with distilled water twice. For color development, tissues were incubated in 0.2% working solution of gold chloride for 10 min and immediately rinsed 10 times with distilled water. After the washes, samples were incubated in 5% sodium thiosulfate for 5 min to fix silver and rinsed in running water for 1 min. After silver fixation, nuclear fast red was used for counterstaining, and tissues were rinsed in running water for 1 min. Ratio of positive cell over total cell was analyzed by QuPath software. Total cells and positive cells were counted by cell counting tool, and ratio was carried out by positive cell over total cell. All measurements were performed in triplicate.

### 2.9. Statistical Analysis

Data are expressed as the mean ± standard error of the mean (SEM) of three or more independent experiments. The statistical significances were examined by Student’s *t*-test. *p*-value < 0.05 was considered statistically significant.

## 3. Results

### 3.1. Effect of 5G EMR on Melanin Contents and Cell Viability of Murine Melanoma Cell, B16F10

**Figure 1:**
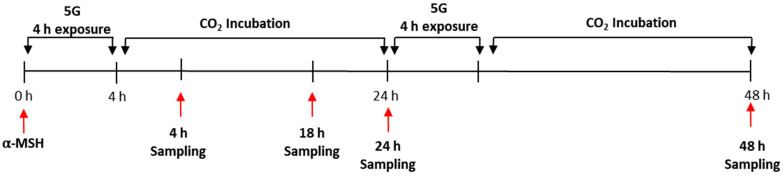
Schematic representation of the schedule of the skin pigmentation study.

**Figure 2:**
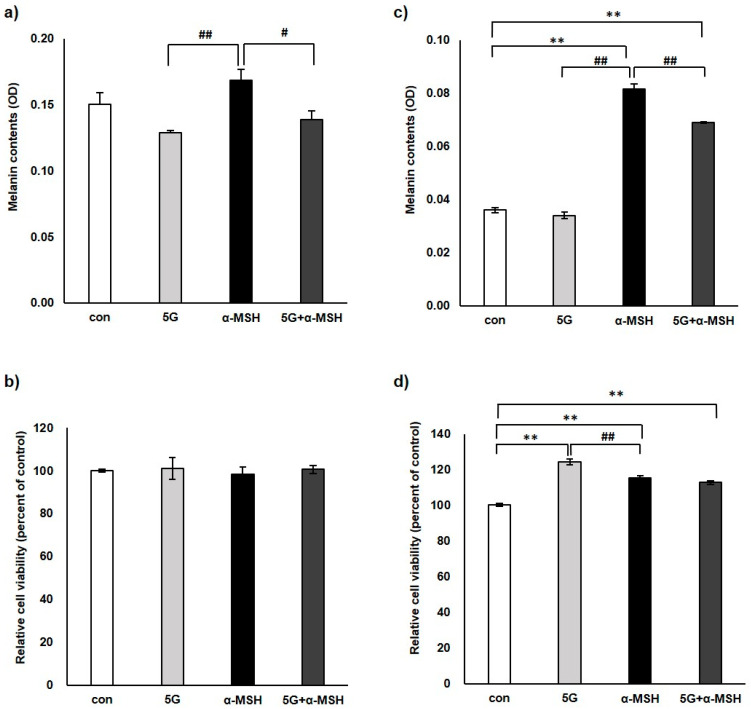
Effects of 5G on melanin contents and cell viability. The murine melanoma cell line, B16F10, was exposed to 5G (28 GHz) for 4 h per day either 5G alone or after a-MSH (0.2 μM) treatment, and then the cells were incubated in CO_2_ incubator at indicated incubation time. (**a**,**c**) Melanin contents were observed by NaOH assay. (**b**,**d**) Cell viability was evaluated by 3-(4,5-dimethylthiazol-2-yl)-2,5-diphenyltetrazolium bromide (MTT) assay. Data are presented with the mean ± SE (*n* = 4) ** *p* < 0.01 compared with the control group; ## *p* < 0.01 and # *p* < 0.05 compared with the α-MSH-treated group.

To investigate the effect of 5G EMR on melanogenesis, murine melanoma cells, B16F10, were exposed to 5G (28 GHz) EMR at the power intensity of 10 W/m^2^ for 4 h per day simulating the normal smartphone use condition either alone or after α-melanocyte stimulating hormone (α-MSH, 0.2 μM) treatment. α-MSH was used to induce melanin synthesis in all experiments (Figure 1). First, B16F10 cells were exposed to 5G EMR for 4 h per day alone or co-exposed with α-MSH, and then the cells were post-incubated in CO_2_ incubator for 24 h. Melanin contents of 5G exposed-B16f10 cells were evaluated and the cell viability was assessed by a formazan-based assays, MTT (Figure 2). The results indicate that 5G decreased α-MSH stimulated melanin synthesis slightly (Figure 2a,c). The cell viability, as measured with MTT, was confirmed not to be reduced by 5G EMR (Figure 2b,d).

### 3.2. Effect of 5G EMR on Cell Morphology of Melanoma Cell, B16F10

**Figure 3:**
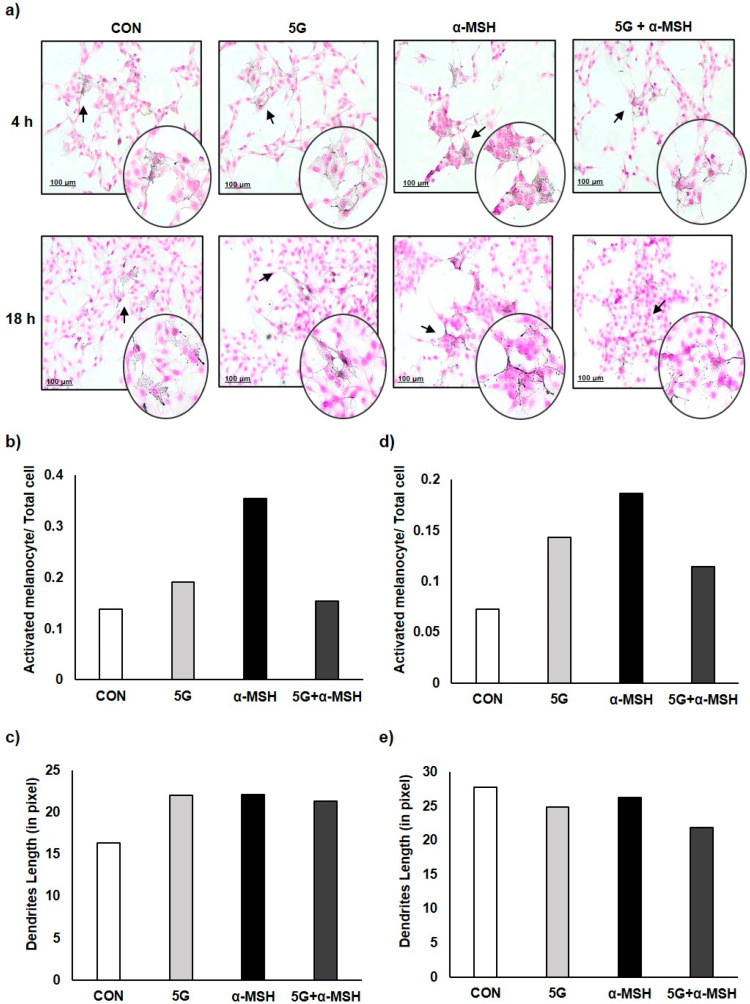
Effects of 5G on the dendrite formation in B16F10 cells. (**a**) Morphological changes were observed under optical microscopy (100×) after Fontana–Masson staining (melanin is stained dark black). B16F10 cells were exposed to 5G (28 GHz) for 4 h per day after a-MSH (0.2 μM) treatment, and then incubated in CO_2_ incubator for the indicated time. Ratio of activated melanocytes over total cell was analyzed by QuPath software, (**b**) 4 h incubation, (**d**) 18 h incubation. Dendrites lengths (in pixels) were analyzed by straight lines in Image J (**c**) 4 h incubation, (**e**) 18 h incubation. (*n* = 3).

The microscopic observation of B16F10 cells after Fontana–Masson (FM) staining, which stains melanin dark black, indicated that melanin synthesis and dendrite formation were inhibited by co-exposure of 5G EMR and α-MSH after 4 and 18 h post-incubation. These data confirmed the results of the melanin assay above that the co-exposure of 5G can attenuate α-MSH stimulated melanocyte activation (Figure 3).

### 3.3. Effect of 5G EMR on mRNA Levels of Melanogenic Enzymes

**Figure 4:**
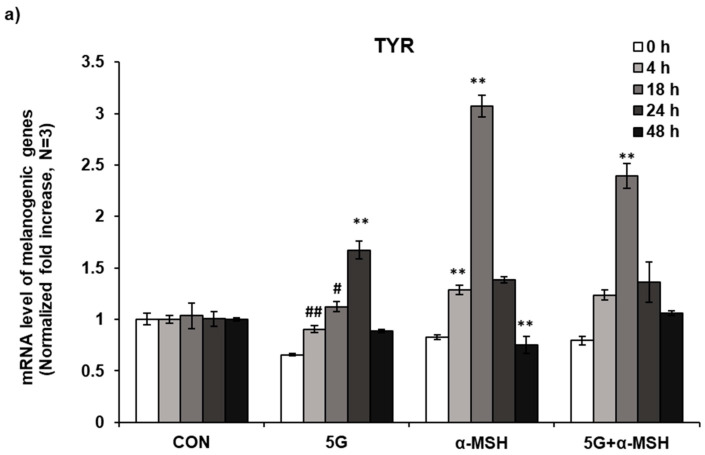

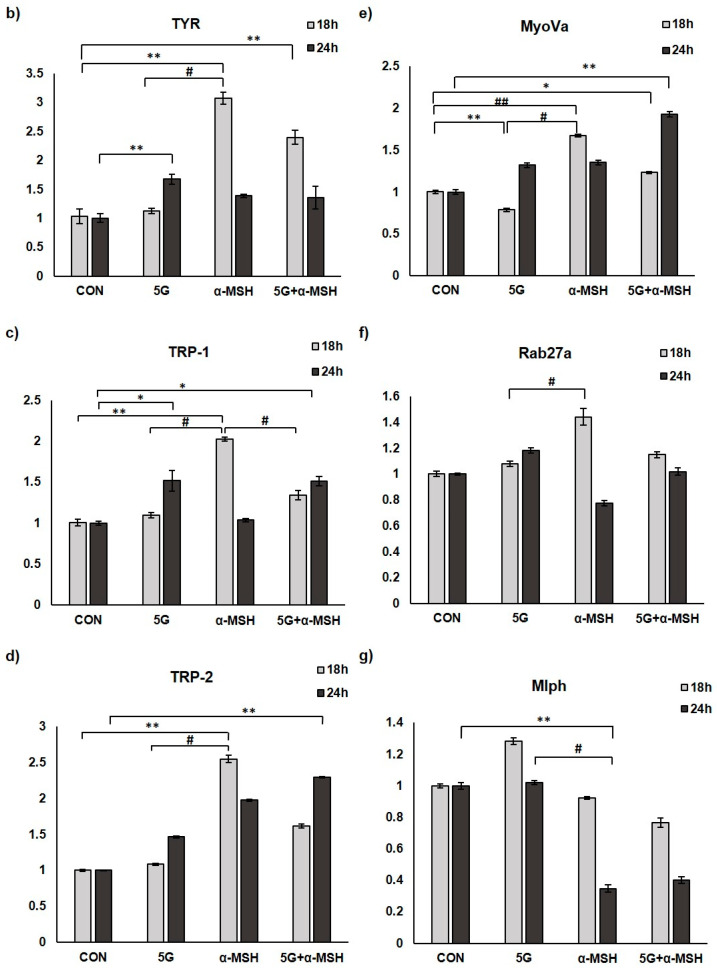
Effects of 5G on mRNA levels of melanogenic genes and melanosome transport. B16F10 cells were exposed to 5G (28 GHz) for 4 h per day either 5G alone or after a-MSH (0.2 μM) treatment, and then cells were incubated for the indicated time. (**a**,**b**) Data indicate mRNA expression level of Tyrosinase according to incubation time. (**c**) Tyrosinase-related protein-1 (TRP-1), (**d**) Tyrosinase-related protein-2 (TRP-2), (**e**) MyoVa, (**f**) Rab27a, and (**g**) Mlph. Data are presented with the mean ± SE (*n* = 3) ** *p* < 0.01 and * *p* < 0.05 compared with the control group; ## *p* < 0.01 and # *p* < 0.05 compared with the α-MSH-treated group.

Tyrosinase (TYR) is the most important and rate-limiting enzyme in melanogenesis, so the downregulation of TYR may ultimately reduce the melanogenesis. The change in expression of TYR mRNA level was determined by real-time polymerase chain reaction (RT-PCR). The data showed that TYR expression was increased by more than two-fold at 18 h after α-MSH treatment, whereas the co-exposure of 5G resulted in decreased expression of TYR (Figure 4a). Exposure of 5G EMR alone slightly induced TYR expression at 24 h, but it was not pronounced compared to α-MSH-induced TYR expression. mRNA expression levels of tyrosinase gene family; TYR, Tyrosinase-related protein 1 (TRP-1), and Tyrosinase-related protein 2 (TRP-2), which are pivotal to melanin synthesis and skin pigmentation [24] were compared. As a result, the upregulated expression levels of TYR, TRP-1, and TRP-2 by α-MSH at 18 h were decreased by the co-exposure of 5G EMR. Interestingly, after 24 h incubation, those effects disappeared (Figure 4b–d).

Rab27a, melanophilin (Mlph), and MyoVa form a tripartite complex that is essential for proper distribution and transfer of melanosomes. Mlph serves as a linker protein between Rab27a and MyoVa, contributing to the stabilization of melanosome structure [25,26,27,28,29,30]. When the expression levels of these molecules were examined, significant but only slight increases in the expression of MyoVa, Rab27a, and Mlph after 5G EMR were observed, suggesting that 5G EMR did not induce significant effects on melanosome transport (Figure 4e–g).

### 3.4. G EMR Exposure Suppresses ROS Production in α-MSH Stimulated B16F10

**Figure 5:**
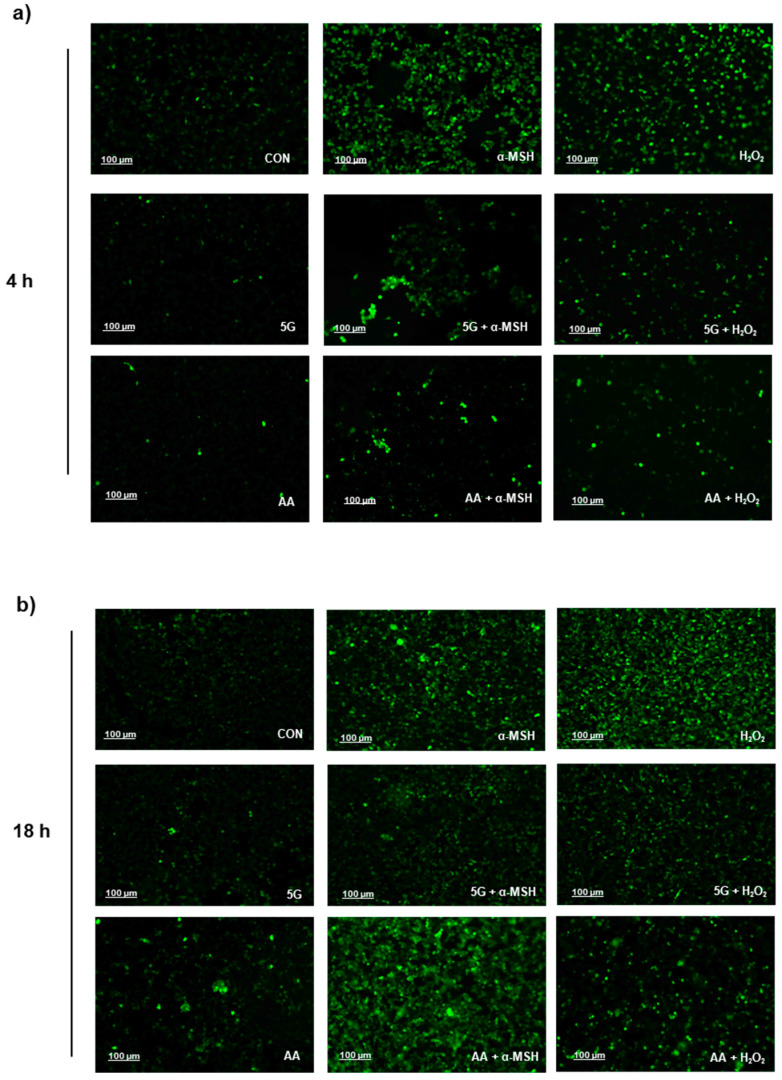
Effects of 5G on ROS generation in 5G exposed B16F10 cells. ROS generation was detected with DCF-DA fluorescence, (**a**) 4 h incubation, (**b**) 18 h incubation. B16F10 cells were exposed to 5G (28 GHz) for 4 h per day after a-MSH (0.2 μM) treatment, and then incubated in CO_2_ incubator on indicated time. An amount of 30 μM of ascorbic acid (AA) was used as positive control for this experiment.

Melanin synthesis accompanies oxidation reactions and superoxide anion (O_2_-) and hydrogen peroxide (H_2_O_2_) generation [31,32]. Tyrosinase oxidizes tyrosine to DOPA, and DOPA to dopaquinone. Dopaquinone is converted into dopachrome through a redox exchange, which is further processed to either dihydroxyindole (5,6-DHI), which is oxidized into indole quinone, or dihydroxyindole carboxylic acid (5,6-DHICA), which is converted into the corresponding quinone [33]. The redox cycling from indoles to quinones generates reactive oxygen species (ROS) that can react with endogenous nucleophiles [34]. Given that melanogenesis is an oxidative process, various kinds of antioxidants from natural sources are widely employed as skin brightening agents [35]. To determine whether 5G EMR can affect the ROS generation, intracellular ROS were monitored by DCF-DA method (Figure 4a). Hydrogen peroxide (H_2_O_2_) was used as positive control for this study. As a result, 5G EMR could suppress both α-MSH and H_2_O_2_ induced ROS production significantly (Figure 5), which may explain the anti-melanogenic effects of 5G EMR. In this experiment, a well-known antioxidant, ascorbic acid (AA), was used as a positive control [36].

### 3.5. Effects of 5G on the Pigmentation in a Pigmented Human Skin Model, Melanoderm™

**Figure 6:**
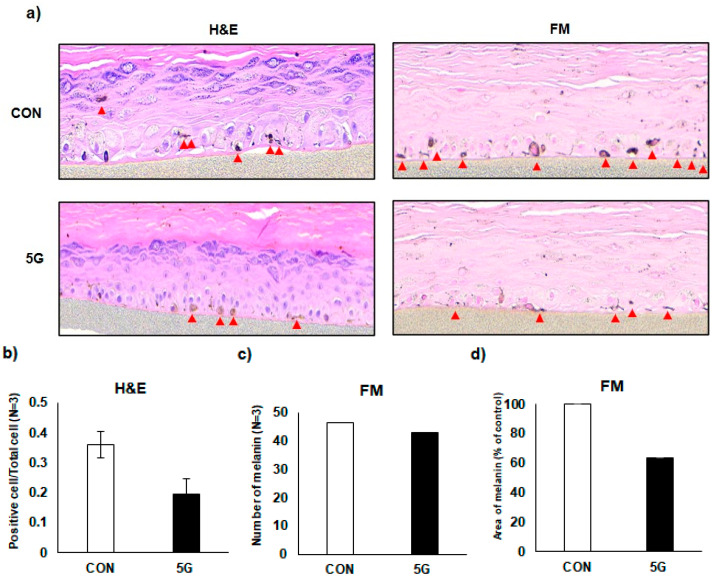
Effects of 5G on Melanoderm™, an artificial pigmented skin model. (**a**) Hematoxylin and eosin (H&E) and Fontana–Masson (FM)-stained tissues. Red arrow heads indicated the stained melanocytes, (**b**) quantitative analysis of H&E-stained tissue, and quantitative analysis of FM-stained tissue by QuPath 0.3.2 software for (**c**) number and (**d**) area of melanin.

An artificial human pigmented epidermis model, Melanoderm™, (MatTek, Ashland, MA, USA), composed of normal human keratinocytes and normal melanocytes, was used to confirm the anti-melanogenic effect of 5G EMR. Before use, Melanoderm™ was stabilized in a 37 °C CO_2_ incubator overnight and then exposed to 5G EMR every other day for 14 days. On the last days of the experiment, the tissues were fixed and stained with hematoxylin and eosin (H&E) or Fontana–Masson (FM). The immunohistochemistry (IHC) data showed that 5G exposure attenuated melanosome accumulation and dendrite formation of melanocytes in the basal layer of Melanoderm™, which was comparable to the 5G unexposed tissue (Figure 6).

## 4. Discussion

Here we demonstrated that 5G (28 GHz) EMR exposure of a normal smartphone use condition attenuates α-MSH stimulated melanin contents in murine melanoma cell line, B16F10 without any appreciable cytotoxicity to the cells. Anti-melanogenic effects of 5G EMR were further confirmed in the morphological examination of the cells and a human pigmented epidermis model, Melanoderm™. Consistently, the expression levels of TYR, TRP-1, and TRP-2 stimulated by α-MSH were attenuated by 5G EMR. Most notably, we demonstrated that 5G exposure effectively suppressed the generation of intracellular ROS in B16F10 cells, suggesting that the ROS-quenching effects of 5G EMR may be involved in its anti-melanogenic effects.

Skin pigmentation has been an important topic related to the health effects of electromagnetic fields (EMF). According to a previous study, skin pigmentation in zebrafish was induced by pulsed electromagnetic fields (PEMFs) (60 Hz) through increasing the activity of TRP-1, which is mediated by the phosphorylation of ERK and p38 [37], reflecting how melanin synthesis and melanocytes may be affected by EMFs [38]. Also, the exposure to extremely low-frequency (ELF) EMFs at low intensities can stimulate melanogenesis in melanocytes [39]. However, it is unclear whether millimeter waves, including 5 G EMR, can affect skin pigmentation, something that may be attributable to the lack of a well-characterized 5G exposure system.

In our previous report, we demonstrated that both LTE and 5G EMRs may not cause skin pigmentation [18] in contrast to EMFs with other bandwidth. However, mRNA levels of melanogenic genes showed slight changes by LTE and 5G while macroscopic skin pigmentation was not observed. Thus, in this present study, we proceeded with research to confirm the effect of melanin synthesis when 5G and α-MSH were exposed at the same time.

We demonstrated that 5G EMR can attenuate melanogenesis through suppressing ROS generation. ROS production in the skin can be caused by various factors, such as UV radiation and air pollution. The production of ROS in the skin is a major cause of skin damage, including hyperpigmentation, wrinkles, rough texture, and aging [40,41]. In particular, ROS regulates melanin synthesis through multiple steps of melanogenesis, which is an oxygen-dependent process. During melanogenesis, the oxidation of L-Dopa to dopaquinone results in the production of superoxide (O_2_^•−^), and the oxidation of the eumelanin precursors DHI and DHICA is associated with hydroxyl radical (H_2_O_2)_ generation [31,42]. Since ROS plays an important role in melanin synthesis, the scavenging of ROS by, for example, antioxidant systems, may attenuate hyperpigmentation or decrease melanin production [43]. Some well-known whitening ingredients, such as glabridin, mulberry, procyanidins, and gingko, have been reported to have antioxidant properties through ROS scavenging as well as whitening effects [44]. Ascorbic acid (Vitamin C), which is a common whitening ingredient, also acts as an ROS scavenger by donating electrons to neutralize free radicals [45,46]. Furthermore, vitamin E (α-tocopherol, α-Toc) has both antioxidant property and depigmenting effect through either scavenging ROS or increasing glutathione [47]. In a study using nanomaterials, graphene oxide nanoribbons (GONR) have been identified as potential agents for a novel antioxidant and skin lightening cosmetic ingredient by chelating metal ions to scavenge free radicals [48]. In this regard, it is highly plausible that 5G EMR may inhibit melanin production through suppressing α-MSH-induced ROS.

Recently, low-level laser therapy (LLLT) has been used in many clinical fields, and in particular, photobiomodulation therapy (PBMT) has become a representative LLL therapy [49,50]. PBMT is a nonthermal therapy that utilizes non-ionizing light sources, such as lasers, light emitting diodes (LEDs), and/or broadband light, falling in the visible (400–700 nm) and near-infrared (700–1100 nm) electromagnetic spectrum [51,52]. Studies have demonstrated the positive effects of PBMT in improving wound healing and preventing chronic ulcers in the skin [53,54,55]. Some studies have demonstrated that irradiation with a weak light at a specific wavelength could promote scavenging of intracellular ROS, alleviate oxidative stress, and improve several pathological conditions [56,57,58]. Indeed, Shon et al. have reported that LED irradiation (635 nm) had antioxidant effects owing to its radical-scavenging properties and modulation of relevant signaling pathways [49]. Moreover, it was also reported that the near-infrared radiation (880 nm) promoted healing with skin damage [59]. Thus, 5G mmW exposures, whose wavelength range is 10 to 1 mm, are likely to possess effects comparable to those obtained from the shorter wavelength lights in the previous studies.

## 5. Conclusions

In summary, the brightening effects of 5G EMR on the skin pigmentation were confirmed at multiple levels ranging from B16F10 cell line and an artificial human pigmented skin model as determined by reduced melanin content, and morphological regression of melanocyte activation. We can observe that 5G exposure attenuated melanin production by regulation melanogenic genes and ROS production. As compared with our previously published study, 5G exposure alone did not affect melanin synthesis, however co-exposure with melanin synthesis stimuli, such as α-MSH, showed an effect of suppressing α-MSH induced melanin as reported in the PMBT studies of the shorter wavelengths. Moreover, it is necessary to examine the effects of 5G EMR on melanin synthesis under more extreme exposure scenarios, such as stronger intensity or for a prolonged time, in the future.

## Data Availability

The data presented in this study are available on request from the corresponding author. The data are not publicly available since they are raw data.
